# Supplementary material for: BCFtools/csq: haplotype-aware variant consequences
Source: Bioinformatics. 2017 Feb 16;33(13):2037–9. doi: 10.1093/bioinformatics/btx100 (PMC5870570; doi:10.1093/bioinformatics/btx100)
Supplement: Supplementary Data [file btx100_supp.zip › supplementary-material.pdf]

Supplementary Material

S1 Comparison and evaluation data

We downloaded two datasets for comparison and evaluation.

- **NA12878**: The [Cleary *et al.*, 2014] segregation-phased NA12878 data was downloaded from the below URL. Only the 4,452,828 sites labelled as `FILTER=PASS` were used.

  `https://s3-us-west-2.amazonaws.com/10x.files/samples/genome/NA12878_WGS/NA12878_WGS_phased_variants.vcf.gz`

- **1000 Genomes**: The 1000 Genomes Phase 3 (v5a) haplotype data was downloaded from the below URL. Evaluations were performed on the the autosomes, chr1-22.

  `ftp://ftp.1000genomes.ebi.ac.uk/vol1/ftp/release/20130502/`

S2 Differences between BCFtools/csq and VEP

With BCFtool/csq run in localized mode on the NA12878 data above, only 11 out of 1.6M predictions differed within coding regions. Six cases were due to ambiguity of insertions occurring at a splice site, which can be interpreted either as part of the exon or outside of it. In these cases, VEP called a frameshift whereas BCFtools called splice acceptor or donor. Note that these all occurred in extremely short introns (2-3bp) bringing into question the accuracy of gene predictions for these transcripts. Another 4 cases were incorrect predictions by VEP, for example a missense event in incomplete CDS misclassified as "start lost", and 1 difference was due to an ambiguous alignment of a 14bp deletion in a repeat region.

Details of these differences are given in the supplementary file `VEP-vs-BCFtools.txt`. The script used to determine these differences is the supplementary file `diff-bt-vep`.

S3 Compound variants in NA12878 and 1000 Genomes

Because one site can be part of multiple compound variants and each can have multiple consequences for different transcripts, we count the number of modified consequence predictions as follows: for each site we take all unique compound variants and split each into its constituents, comparing the most severe compound prediction with the most severe localized prediction.

In haplotype-aware mode, on the NA12878 data above, BCFtools/csq found 18 frame-recovered indels with altered sequence shorter than 30bp, 12 stop gains recovered as missense events, and 3 novel stops.

The list of all compound variants found in NA12878 is given in the supplementary file `cmpnd.NA12878.txt` and the list of all compound variants found in the 1000 Genomes dataset is given in the supplementary file `cmpnd.1000GP.txt.gz`. These are summarised in Table 1 and Suppl. Fig. 1. The script used to generate these lists of compound variants is the supplementary file `prn-cmpnd`.

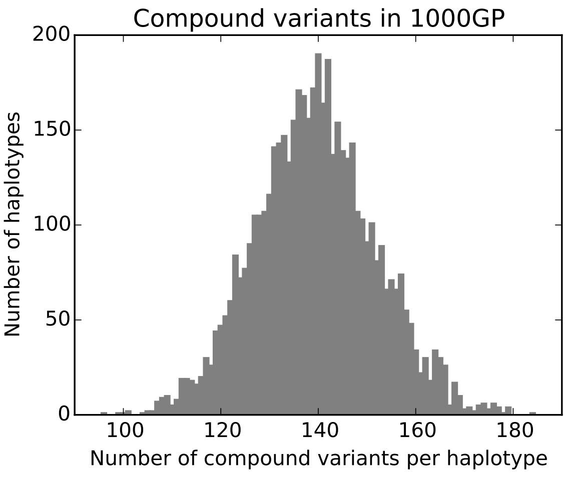

Figure S1: Number of compound variants in 1000 Genomes Project data.

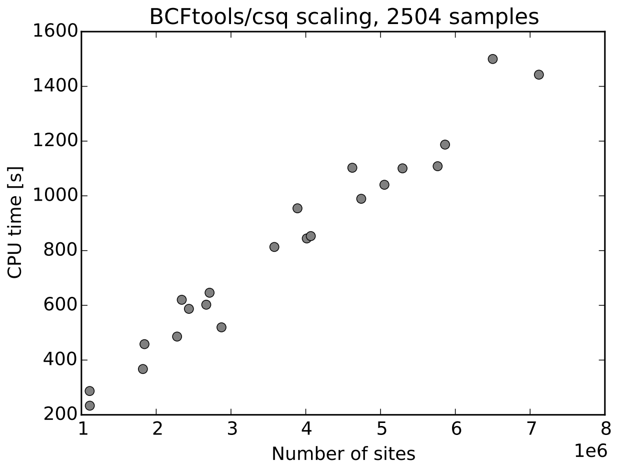

Figure S2: Linear scaling of BCFtools/csq with the number of sites. Tests were performed on 1000 Genomes Phase 3 data.

S4 Error rate in 1000 Genomes compound variants

Given that the typical switch error in statistically phased haplotypes is $\epsilon = 0.01$, the compound error rate can be estimated from the distribution of heterozygous genotypes in compound variants (Suppl. Fig. S6) as

$$\frac{1}{N}\sum_{i=1}^{N}\sum_{k=0}^{n_i/2}\binom{n_i}{2k+1}\epsilon^{2k+1}(1-\epsilon)^{n_i-2k-1},$$

where N is the number of compound variants and n_i is the number of heterozygous genotypes within the i-th variant. The sum over k and the binomial term select combinations with odd number of switch errors.

S5 Performance and scaling

Table S1: Performance comparison of BCFtools/csq with three popular consequence callers using a single-sample VCF with 4.5M sites. Note that at the time of writing, a new version of VEP is being prepared which brings the running time down and makes the performance of VEP comparable to the other programs (William McLaren, personal communication).

	VEP	snpEff	ANNOVAR	csq (local)	csq (haplotype)
CPU time	6 hrs	35 min	24 min	101 sec	92 sec
Memory	17 GB	7.8 GB	3.9 GB	207 MB	208 MB

The commands and versions used in the above evaluation are listed below:

VEP (v82)

```
perl variant_effect_predictor_v82.pl --db_version 82 -t SO --format vcf --cache \
  --dir vep_cache --offline --species human --symbol --biotype --vcf --no_stats \
  --assembly GRCh37 --no_progress --quiet
```

snpEff (v4.2)

```
java -Xmx14g -jar snpEff.jar -noStats -noLof -noInteraction -noNextProt \
  -noMotif GRCh37.75
```

ANNOVAR (2016Feb01)

```
table_annovar.pl --vcfinput --tempdir tmp --outfile NA12878.annovar --buildver hg19 \
  --protocol ensGene --operation g NA12878.vcf annovar/humandb
```

BCFtools/csq (local) (v1.3.1-179-gd7f6692)

```
bcftools csq -f hs37d5.fa -g GRCh37.82.gff3.gz -l -s -
```

BCFtools/csq (haplotype) (v1.3.1-179-gd7f6692)

```
bcftools csq -f hs37d5.fa -g GRCh37.82.gff3.gz -p s -n 64
```

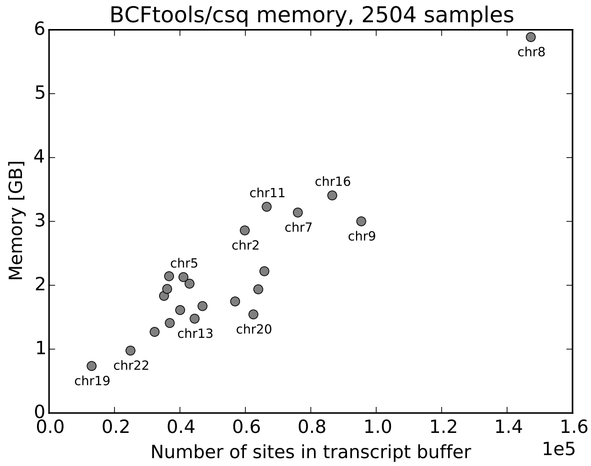

Figure S3: The maximum amount of memory required by the BCFtools/csq haplotype-aware calling is linear with the number of sites that needs to be kept in memory before flushing the transcript. Tests were performed on 1000 Genomes Phase 3 data.

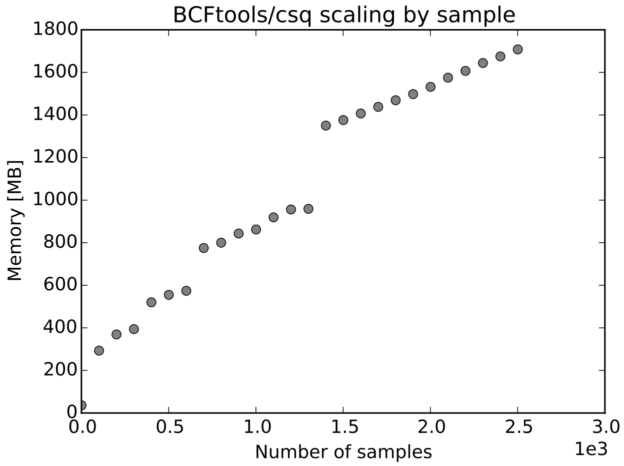

Figure S4: Linear scaling of memory required by BCFtools/csq with the number of samples. Tests were performed on 1000 Genomes Phase 3 data, chromosome 1.

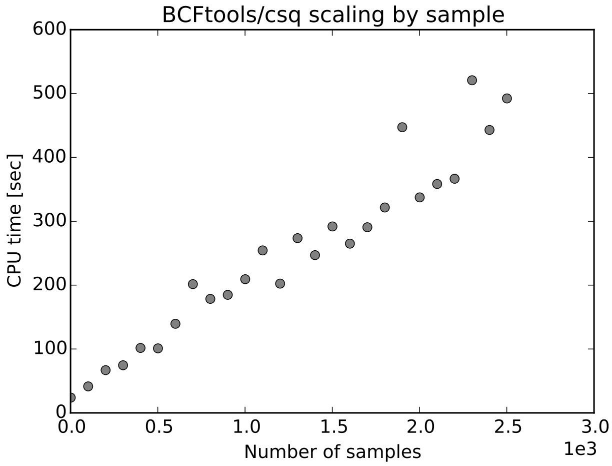

Figure S5: The CPU time by the number of samples. Tests were performed on 1000 Genomes Phase 3 data, chromosome 1. Localized mode took 28 seconds, while running in haplotype-aware mode on all 2,504 samples took 492 seconds.

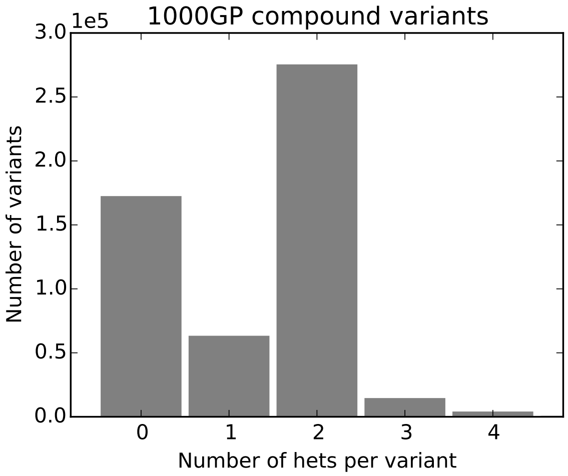

Figure S6: The distribution of heterozygous genotypes in compound variables in 1000 Genomes Phase 3 data.

References

[Cingolani *et al.*, 2012] Cingolani P, *et al.* (2012) A program for annotating and predicting the effects of single nucleotide polymorphisms, SnpEff, *Fly (Austin)*, **6(2)**, 80-92.

[Cleary *et al.*, 2014] Cleary JG, *et al* (2014) Joint variant and de novo mutation identification on pedigrees from high-throughput sequencing data, *J. Comput. Biol.*, **21(6)**, 405-419.

[Lek *et al.*, 2016] Lek M, *et al.* (2016) Analysis of protein-coding genetic variation in 60,706 humans, *Nature*, **536(7616)**, 285-91.

[Loh *et al.*, 2016] Loh P, *et al.* (2016) Reference-based phasing using the Haplotype Reference Consortium panel, *Nat. Genetics.*, **48(11)**, 1443-1448.

[McCarthy *et al.*, 2014] McCarthy D, *et al.* (2014) Choice of transcripts and software has a large effect on variant annotation, *Genome Med.*, **6(3)**, 26.

[McCarthy *et al.*, 2016] McCarthy S, *et al.* (2016) A reference panel of 64,976 haplotypes for genotype imputation, *Nat. Genetics.*, **48(10)**, 1279-83.

[McLaren *et al.*, 2016] McLaren W, *et al.* (2016) The Ensembl Variant Effect Predictor, *Genome Biol.*, **17(1)**, 122.

[Sharp *et al.*, 2016] Sharp K, *et al.* (2016) Phasing for medical sequencing using rare variants and large haplotype reference panels, *Bioinformatics.*, **32(13)**, 1974-80.

[Wang *et al.*, 2010] Wang K, Li M, Hakonarson H (2010) ANNOVAR: functional annotation of genetic variants from high-throughput sequencing data, *Nucleic Acids Res.*, **38(16)**, e164.

[Wei *et al.*, 2015] Wei L *et al.* (2015) MAC: identifying and correcting annotation for multi-nucleotide variations, *BMC Genomics.*, **16**, 569.

[Zheng *et al.*, 2016] Zheng GX, *et al.* (2016) Haplotyping germline and cancer genomes with high-throughput linked-read sequencing, *Nat. Biotechnol.*, **34(3)**, 303-11.
