# Supplementary figures and images for: BCFtools/csq: haplotype-aware variant consequences
Source: Bioinformatics. 2017 Feb 16;33(13):2037–9. doi: 10.1093/bioinformatics/btx100 (PMC5870570; doi:10.1093/bioinformatics/btx100)

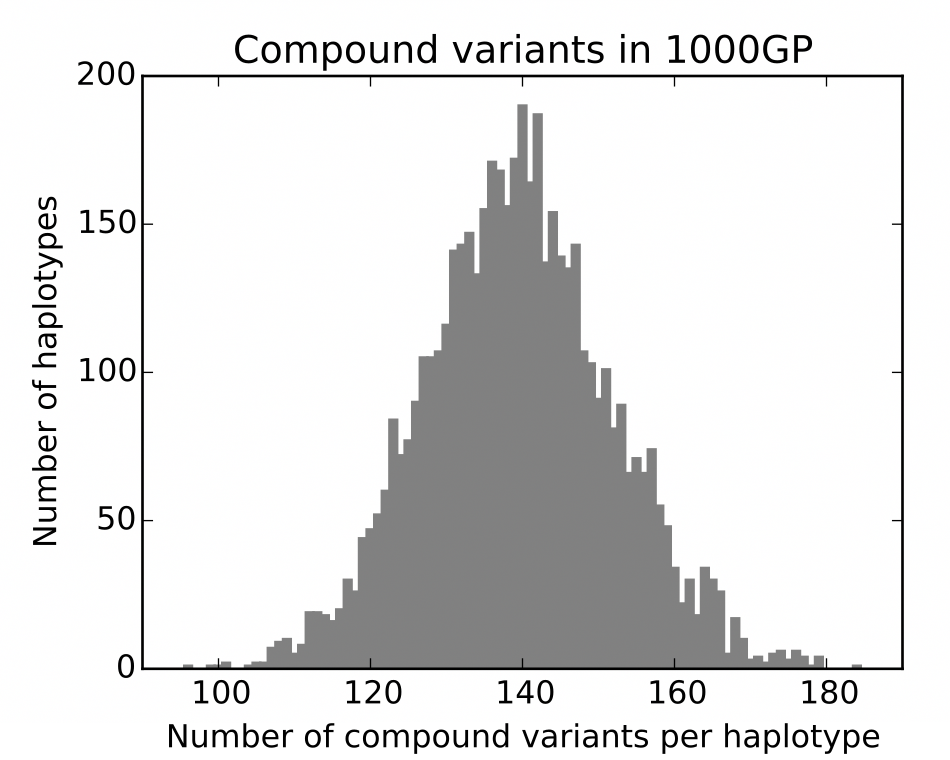

Supplement: Supplementary Data [file btx100_supp.zip › SupplementaryFigure1.png]

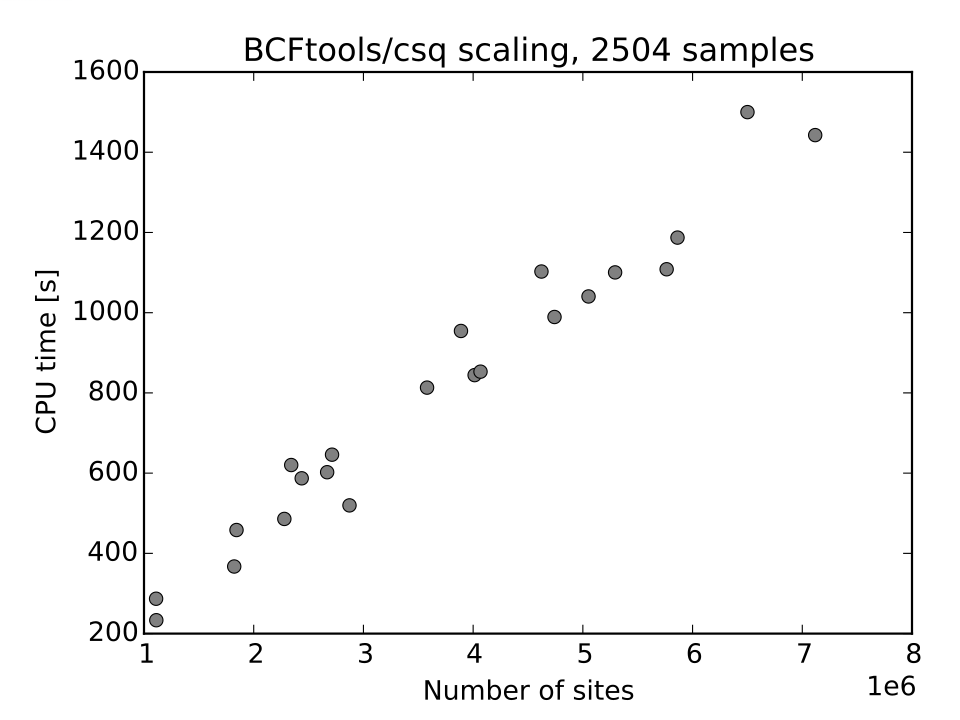

Supplement: Supplementary Data [file btx100_supp.zip › SupplementaryFigure2.png]

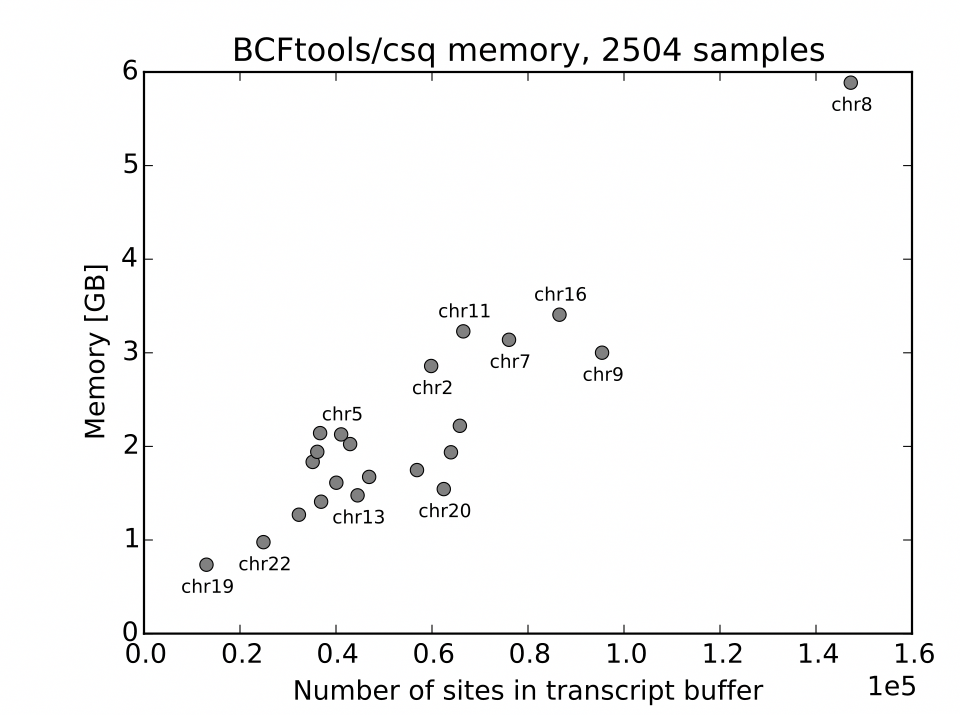

Supplement: Supplementary Data [file btx100_supp.zip › SupplementaryFigure3.png]

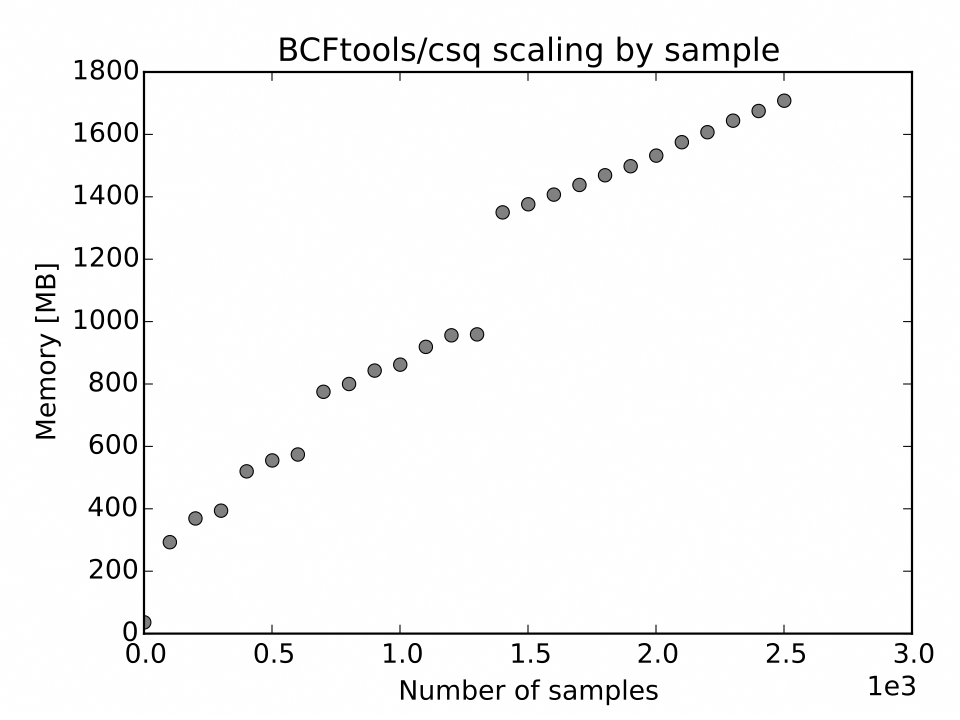

Supplement: Supplementary Data [file btx100_supp.zip › SupplementaryFigure4.png]

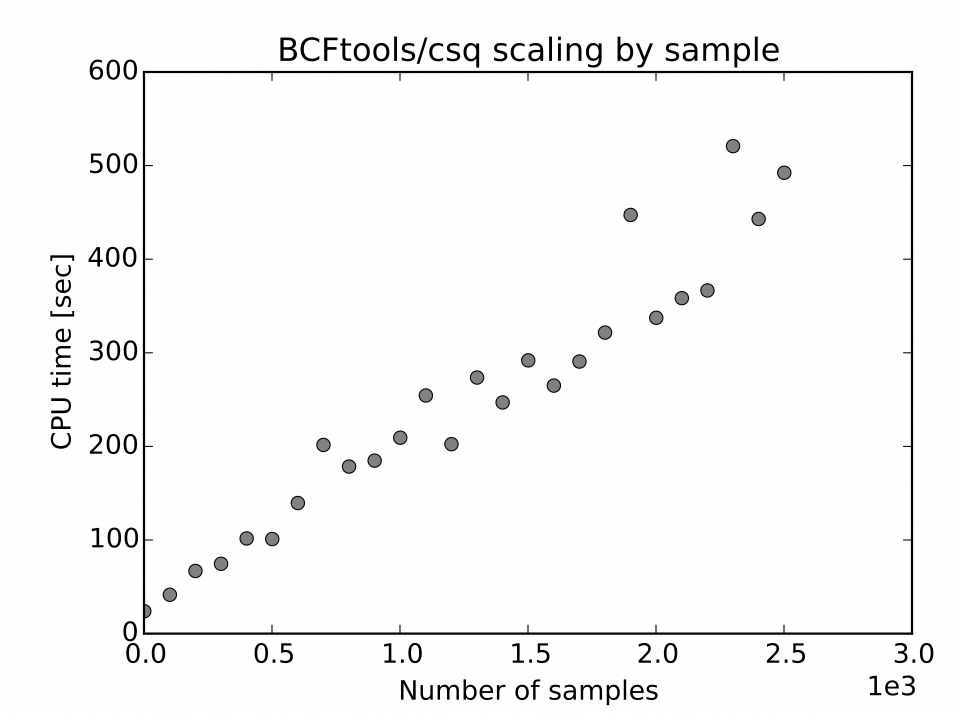

Supplement: Supplementary Data [file btx100_supp.zip › SupplementaryFigure5.png]

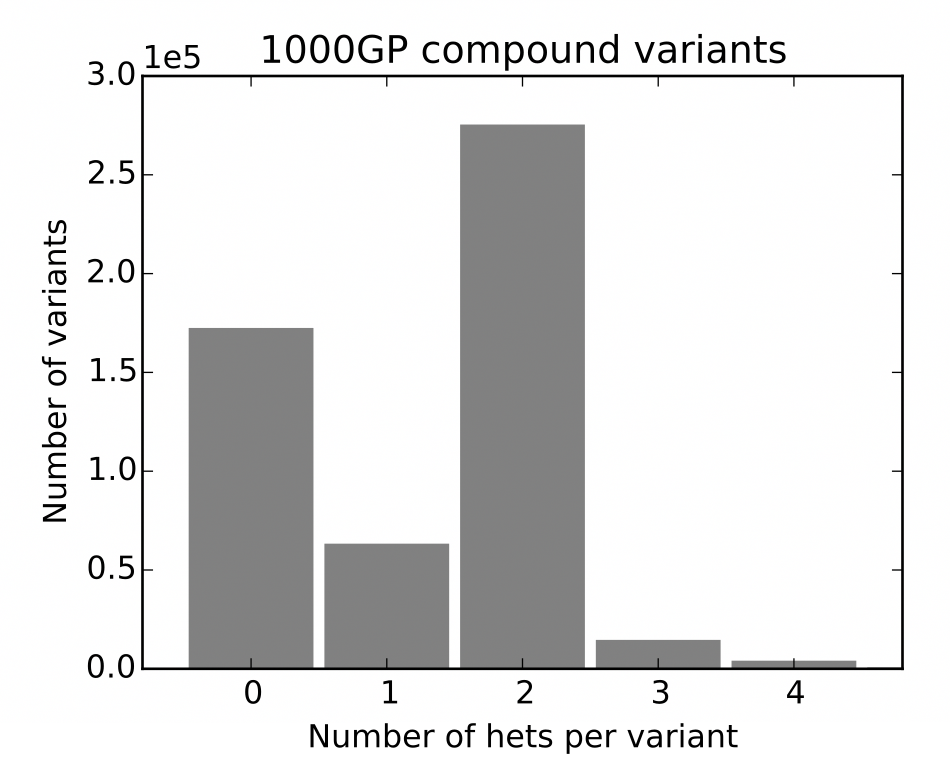

Supplement: Supplementary Data [file btx100_supp.zip › SupplementaryFigure6.png]
